# The prevalence of autosomal dominant polycystic kidney disease (ADPKD): A meta-analysis of European literature and prevalence evaluation in the Italian province of Modena suggest that ADPKD is a rare and underdiagnosed condition

**DOI:** 10.1371/journal.pone.0190430

**Published:** 2018-01-16

**Authors:** Andrea Solazzo, Francesca Testa, Silvia Giovanella, Marco Busutti, Luciana Furci, Paola Carrera, Maurizio Ferrari, Giulia Ligabue, Giacomo Mori, Marco Leonelli, Gianni Cappelli, Riccardo Magistroni

**Affiliations:** 1 Dipartimento Chirurgico, Medico, Odontoiatrico e di Scienze Morfologiche con Interesse Trapiantologico, Oncologico e di Medicina Rigenerativa, Università degli Studi di Modena e Reggio Emilia, Modena, Italy; 2 UO Nefrologia, Dialisi e Trapianto, Dipartimento di Medicina Specialistica, Diagnostica e Sperimentale, Ospedale Sant'Orsola-Malpighi, Alma Mater Studiorum Università di Bologna, Bologna, Italy; 3 Divisione di Nefrologia Dialisi e Trapianto Renale, Dipartimento interaziendale ad attività integrata Malattie Nefrologiche, Cardiache e Vascolari, Azienda Ospedaliero Universitaria di Modena, Modena, Italy; 4 Division of Genetics and Cell Biology, Unit of Genomics for human disease diagnosis, and Laboratory of Clinical Molecular Genetics, San Raffaele Scientific Institute, Milan, Italy; 5 Università Vita e Salute San Raffaele, Milan, Italy; Istituto Di Ricerche Farmacologiche Mario Negri, ITALY

## Abstract

**Background and objectives:**

ADPKD is erroneously perceived as a not rare condition, which is mainly due to the repeated citation of a mistaken interpretation of old epidemiological data, as reported in the Dalgaard's work (1957). Even if ADPKD is not a common condition, the correct prevalence of ADPKD in the general population is uncertain, with a wide range of estimations reported by different authors. In this work, we have performed a meta-analysis of available epidemiological data in the European literature. Furthermore we collected the diagnosis and clinical data of ADPKD in a province in the north of Italy (Modena). We describe the point and predicted prevalence of ADPKD, as well as the main clinical characteristics of ADPKD in this region.

**Methods:**

We looked at the epidemiological data according to specific parameters and criteria in the Pubmed, CINAHL, Scopus and Web of Science databases. Data were summarized using linear regression analysis. We collected patients’ diagnoses in the Province of Modena according to accepted clinical criteria and/or molecular analysis. Predicted prevalence has been calculated through a logistic regression prediction applied to the at-risk population.

**Results:**

The average prevalence of ADPKD, as obtained from 8 epidemiological studies of sufficient quality, is 2.7: 10,000 (CI95 = 0.73–4.67). The point prevalence of ADPKD in the province of Modena is 3.63: 10,000 (CI95 = 3.010–3.758). On the basis of the collected pedigrees and identification of the at-risk subjects, the predicted prevalence in the Province of Modena is 4.76: 10,000 (CI 95% = 4.109–4.918).

**Conclusion:**

As identified in our study, point prevalence is comparable with the majority of the studies of literature, while predicted prevalence (4.76: 10,000) generally appears higher than in the previous estimates of the literature, with a few exceptions. Thus, this could suggest that undiagnosed ADPKD subjects, as predicted by our approach, could be relevant and will most likely require more clinical attention. Nevertheless, our estimation, in addition to the averaged ones derived from literature, not exceeding the limit of 5:10,000 inhabitants, are compatible with the definition of rare disease adopted by the European Medicines Agency and Food and Drug Administration.

## Introduction

Autosomal dominant polycystic kidney disease (ADPKD) is recognized as the most frequent Mendelian kidney disease. In Europe, ADPKD is the fourth diagnosis for both the incidence and prevalence of renal diseases that require replacement therapy [1] and 1 in 10 patients needing renal replacement therapy has ADPKD [2]. The predominant phenotype of ADPKD is the accumulation of cysts in renal parenchyma, however, a number of other events accompany the condition such as cysts in other organs, (liver, pancreas, spleen, seminal vesicles, and arachnoid membrane), cardiovascular abnormalities (intracranial aneurysms, aortic root dilatation and aneurysms, mitral valve prolapse), abdominal wall hernias and other rarer phenotypes (epididymal cysts, etc.). The condition is genetically heterogeneous and is caused by the mutation of two polycystin genes, PKD1 and PKD2 [3, 4] and much more rarely by two other recently identified genes: GANAB [5], PMM2 [6]. The genetic defect of ADPKD subverts the normal differentiated phenotype of renal tubular epithelium. Cyst accumulation and growth replaces normal kidney parenchyma in a complex process that is accompanied by fibrosis and interstitial inflammation.

The research question posed by this study concerns the prevalence of ADPKD in European territory. A correct understanding of the epidemiology of a condition is central to many aspects of the health organization and clinical research concerning the same condition (e.g., a program of clinical trials, access to orphan drugs designation, the implementation of specific emerging treatments, the evaluation of the performance of treatment, etc.). In the past, in the introduction of their works, many authors have frequently reported a prevalence of ADPKD between 1/400 and 1/1000, thus referring to Dalgaard’s seminal work[7]. In fact, Dalgaard did not ascertain the point prevalence of ADPKD but rather estimated a morbid risk—the theoretical risk of being ill from ADPKD during a lifetime of 80 years duration. Dalgaard’s paper has long been misunderstood, and based on this equivocation, ADPKD was incorrectly considered as a peculiarly frequent condition in spite of being a genetic disorder. In ADPKD, available epidemiological estimates are conflicting and prevalence is uncertain. The evaluation of the prevalence of ADPKD is challenging, and this difficulty is fully reflected in the broad range of estimations reported by different authors over the years[7–22]. There are different approaches to estimating disease occurrence in a population. The choice of approach will depend on many different factors, such as the amount of patient data available and the accuracy of the result required.

In this paper, we have performed a meta-analysis of the epidemiological data (cohort studies of prevalence of ADPKD in the general population) in the available literature. Furthermore we evaluated the prevalence of ADPKD in a circumscribed geographic region of northern Italy. In particular, we focused on identifying all the affected and at-risk subjects, starting from the pedigrees of the index cases. Furthermore, we present an unprecedented strategy for the management of missing diagnosis in the ADPKD epidemiology. Our analysis allows us to safely confirm that ADPKD can be considered a rare disease, in terms of the criteria required by the European Medicine Agencies (EMA) and Food and Drug Administration (FDA).

## Materials and methods

### Selection criteria of the available literature

Articles in English language (at least in the abstract section, in case of interesting articles with full article in other language than English a translation has been obtained) reporting epidemiologic data of patients affected by ADPKD in the general population of the European Union countries were considered eligible.

We performed searches of four electronic databases in order to identify high-quality epidemiologic studies published on ADPKD: the National Library of Medicine PubMed database, the Web of Science database, the Scopus database and the CINAHL database.

The search used the Medical Subject Heading terms ‘Polycystic Kidney’, Autosomal Dominant’ or ‘Polycystic Kidney Diseases and ‘incidence study’ or ‘prevalence study’ or ‘epidemiologic study’ and encompassed all studies published between January 1980 and February 2017, as ultrasound and other modern diagnostic methods for ADPKD detection were available during this timeframe. The research strategy has been adapted according to the specific query structure of each database. In detail the adopted search strategies are:

NLM PubMed: ((ADPKD OR ("Polycystic Kidney Diseases"[Mesh]) OR "Polycystic Kidney, Autosomal Dominant"[Mesh])) AND ("Epidemiologic Studies"[Mesh]OR "Cross-Sectional Studies"[Mesh] OR "Cohort Studies"[Mesh] OR (prevalence[Title] OR epidemiology[Title]))

Filters: Publication date from 1980/01/01 to 2017/02/28)).

Cinahl: (ADPKD OR MH "polycystic kidney disease+" OR MH "Polycystic Kidney, Autosomal Dominant+") AND (MH "Epidemiologic Study+" OR MH "Cross-Sectional Study+" OR MH "Cohort Study+" OR TI "prevalence" OR TI "epidemiology")

Filters: Publication date from 1980/01/01 to 2017/02/28)).

Scopus: ((ABS(ADPKD) OR ABS(autosomal dominant polycystic kidney disease)) AND ((ABS(epidemiology) OR ABS(Epidemiologic Study) OR ABS(Cross-Sectional Study) OR ABS(Cohort Study))))

Filters: Publication date from 1980/01/01 to 2017/02/28)).

Web of Science: ((TI = (ADPKD) OR TI = (autosomal dominant polycystic kidney disease) OR (TS = (ADPKD) OR TS = (autosomal dominant polycystic kidney disease)) AND ((TI = (epidemiology) OR TI = (Epidemiologic Study) OR TI = (Cross-Sectional Study) OR TI = (Cohort Study) OR (TS = (epidemiology) OR TS = (Epidemiologic Study) OR TS = (Cross-SecTSonal Study) OR TS = (Cohort Study))))

Filters: Publication date from 1980/01/01 to 2017/02/28)).

Further reports were added to the list of available literature following a manual review of the citations from relevant studies.

Criteria used to select relevant studies from the preliminary list included: selection of population-based studies and registry data, epidemiologic reviews, validity studies, studies of clinical characteristics in large patient samples. In addition, an adequate sampling and power analysis, an appropriate denominator for prevalence estimates and a contemporary and largely accepted ADPKD definition were required (according to Pei, Obaji et al. 2009 ^19^, Pei, Hwang et al. 2015 ^20^).

The papers have been reviewed by two independent researchers according to the previous criteria. Data have been extracted in piloted forms. Quality of the studies was assessed by a standardized scale (Newcastle-Ottawa scale). A detailed report of the assessment is reported in the Table I in S1 File of the supplemental material. In the Table A–H in S1 File of the supplemental material a synthetic report of the Newcastle-Ottawa scale is reported for each study. Disagreements between the review authors in particular studies were resolved by discussion, with involvement of a third review author where necessary.

The following data items have been collected from the selected articles: Type of Source, Region, Collection Year(s), Case definition, Data Collection Method, Design, Reference Population, Risk Factors, Incidence, Renal Replacement Therapy, Mortality, Point Prevalence Predicted Prevalence (PrP). These data are tabulated in Table A–H in S1 File of the supplemental materials.

### Identification of affected patients in the province of Modena

The study has been approved by the Ethical Committee of the Province of Modena under the name ‘GREAt’ (GRoup for Epidemiological study in ADPKD). Before clinical evaluation and data collection, patients received adequate information and signed an informed consent form. Data were collected extensively from different sources: Administrative Electronic Databases of Outpatient Clinics and Hospital Admission, Renal Replacement Therapy Registry (http://www.regdial.it/) and Radiologic Databases. Subjects diagnosed with the Renal Cyst condition were singularly reviewed. For Outpatient Clinics of small centers without data capture based on Electronic Databases, the hard copies of medical notes have been reviewed to identify patients with a diagnosis related to ADPKD.

Inclusion criteria for patients were based on imaging evaluation by ultrasound according to Pei et al. [23] and MRI diagnostic criteria, as reported by Pei et al.[24]. Details of the criteria are reported in the Supplemental Materials (Appendix A in S1 File of the supplemental material).

In the uncertain cases where a genetic test with a conclusive identification of a likely pathogenic or pathogenic variant [25] was available, it has been accepted to confirm the diagnosis.

### Strategy for patient identification

1) Searching all the available clinical sources initially allowed the identification of index cases; 2) we then collected pedigree information from the index cases and identified all of the at-risk subjects; 3) finally, we collected the clinical information of index cases and at risk subjects by summoning them for a clinical visit and/or collecting data from available clinical databases (specifically, the radiologic archives).

### Statistical analysis

#### Meta-analysis

Linear regression has been adopted to combine prevalence between the selected epidemiologic studies (Stata/IC 11.2, Stata Corp, Tx, USA). Combined prevalence between studies has been obtained as coefficient of the linear regression of patients against population with 95% confidence intervals. Quantitative assessment of study heterogeneity has been assessed using Cochran’s Q. Q is the weighted sum of squares on a standardized scale. This test has low power to detect heterogeneity and it is suggested to use a value of 0.10 as a cut-off for significance[26]

#### Epidemiologic study

Binary Logistic Regression (Marginal Standardization Method) [27] was used to predict the probability of the occurrence of the event “presence of ADPKD” in the at-risk population. The objective of the analysis was to determine, using logistic regression the relative contribution of independent variables (predictors) according to the intensity of their influence (proven by statistical significance) upon the occurrence of values of the dependent ADPKD risk scores.

The quality of the model was judged by examining the overall significance of the model. This could be done by testing the hypothesis (H_0_:β = 0) which means that all the regressor were insignificant on ADPKD. We have rejected this hypothesis, indicating that the regressors have got significant effect on ADPKD.

By the procedure of curve estimation we have produced regression statistics and a related separate model was produced for each dependent variable. The model also saved the predicted values and the 95% prediction intervals. We have estimated the predicted prevalence rates and the 95% prediction intervals.

We refer readers who wish to apply marginal standardization using SAS (SAS Institute Inc., Cary, NC) to a macro described elsewhere [27]. The PROCRLOGIST command in SAS-callable SUDAAN (Research Triangle Institute, Research Triangle Park, NC) can implement Marginal Standardization.

**Missing data (age).** As recommended by EMA’s guideline on missing data in the case of continuous variables (EMA/CPMP/EWP/1776/99), linear mixed models (LMMs) have been used to impute missing values. The peculiarity of LMMs lies in the way parameters are treated. This model assume that model’s parameter (age) is composed of a fixed term (a mean value common to all individuals) plus a random effect (which conveys the between-subject variability). Affected (with age AND gender, after LMMs procedure) N = (238 + 16) = 254. All the subjects “Affected Clinically Defined” (N = 254) contribute to the Prevalence Rate. The model is assessed by comparing the calculated prevalence with the observed data by gender. Clinically Defined Population (N = 254).

**Age and sex-specific prevalence.** Age and sex-specific prevalence rates of ADPKD were computed as the ratio of the number of cumulative cases identified divided by the population for age and sex-specific groups. To estimate the prevalence of ADPKD we fitted a non-linear regression model, including age (in 5-year groups) and gender. We summarized prevalence rates separately for men and women by estimating an individual’s probability of being diagnosed with ADPKD during her or his lifetime (lifetime risk). This measure is an overestimation of the true lifetime risk, since dying from other causes reduces the cumulative probability of developing ADPKD in a population. This methodology allows the estimation of age-conditional probabilities of developing ADPKD taking into account that individuals dying from causes other than the disease of interest cannot develop that disease in the future (as a consequence, the adjusted lifetime risk is lower than the non-adjusted value).

The distribution of the cumulative risk to be affected according to age and differentiated by sex is represented in Fig A in S1 File of the supplemental material.

**Family Risk Score.** The Family Risk Score was defined as the ratio of affected subjects compared to those at risk in a family. This parameter has been calculated in all the collected pedigrees. The predicting value of this parameter relies on the well documented role of genetic variant in the severity of the condition. This is reported for the locus effect (PKD1 / PKD2 contribution) [28] as well as for the allele effect (role of the type of variant (missense / truncating) especially in PKD1 subjects [29, 30]. By the assumption that a family share the same type of mutation we inferred that there is a familiar risk contribution that we approximated in the Family Risk Score. Fig B in S1 File of the supplemental material represents the distribution of the Family Risk Score in our families. The Family Risk Score has not been calculated in the sporadic cases (absence of family history). In the logistic regression model the Family Risk Score has been adjusted for the percentage of subjects without a family history (15%).

**Genetic analysis.** Genetic analysis for PKD1 and PKD2 were available for a subset of subjects. The test was based on the sequencing of the two genes—whole coding regions and exon junctions—using the Sanger direct method. Methods have been extensively reported in a previous paper of our group [31]. All genetic and phenotypic data have been anonymized and uploaded to an electronic database (LOVD3.0 platform)[32, 33] that is publicly available (https://databases.lovd.nl/shared/genes/PKD1; https://databases.lovd.nl/shared/genes/PKD2). Details on the molecular genetic methods are provided in the Supplemental Materials section.

## Results

### Available epidemiologic data and combined analysis

**Fig 1:**
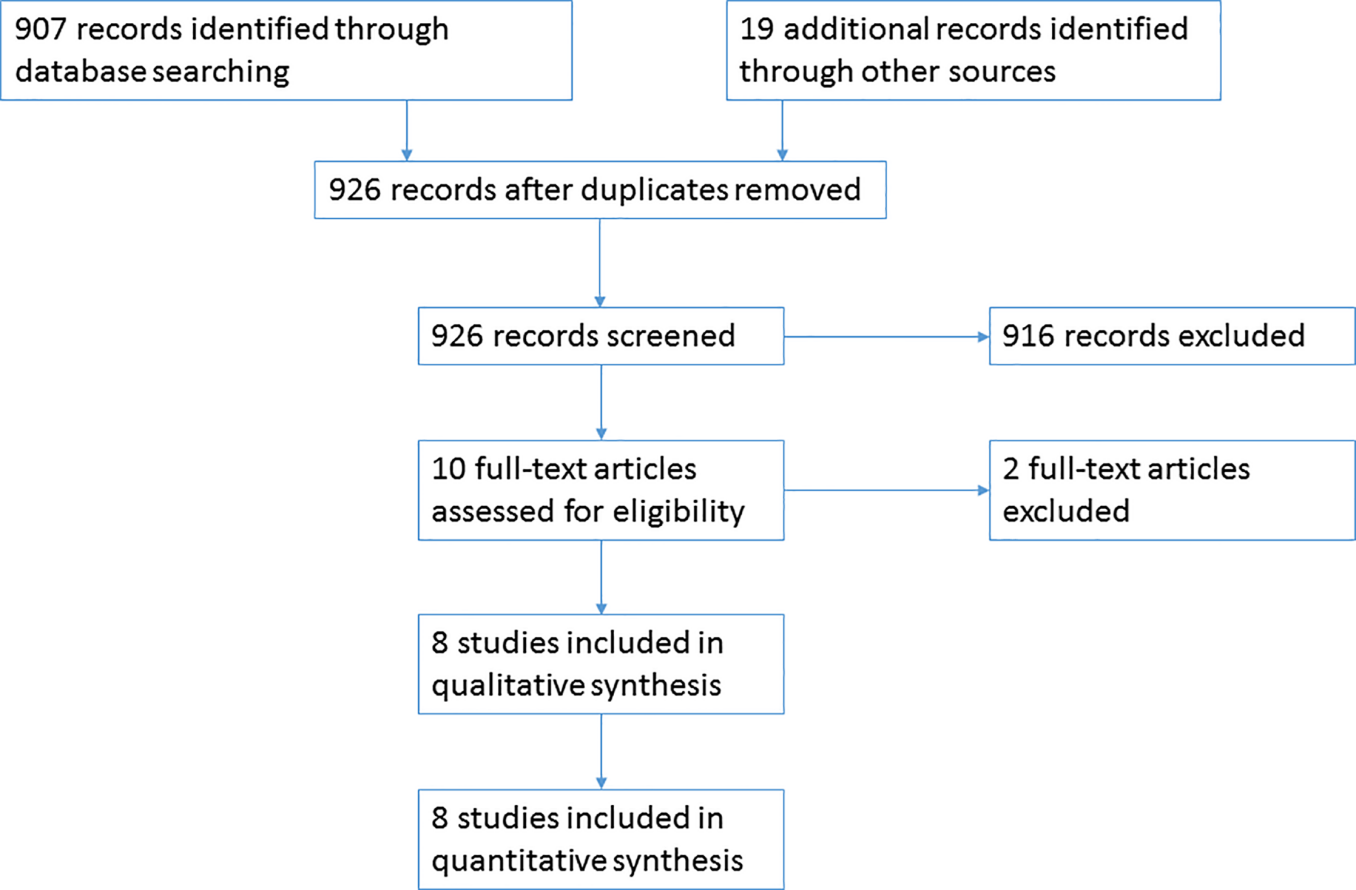
Flow of information through the different phases of the selection of available studies.

We performed an extensive literature research based on a database search and by manually checking the references of the identified articles. In all, 926 citations were identified, of which 916 were excluded according to the selection criteria. In particular, some studies encompassed renal cystic conditions other than ADPKD (e.g., ARPKD or Tuberous Sclerosis Complex), or were otherwise clinical studies based on small or methodologically flawed samples. Of the analyzed ten full-text articles one was excluded because of an unacceptable clinical definition of ADPKD [Heidland, Bahner et al. 2009[11]], the second because of the absence of a reference population [World 2012] [20]). According to these criteria, eight population-based studies have been selected for data analysis. The Prisma [34] flowchart for the selection of the available studies is depicted in Fig 1. The main data of these studies are tabulated (Table A–H in S1 File) in the Supplemental Materials section.

**Fig 2:**
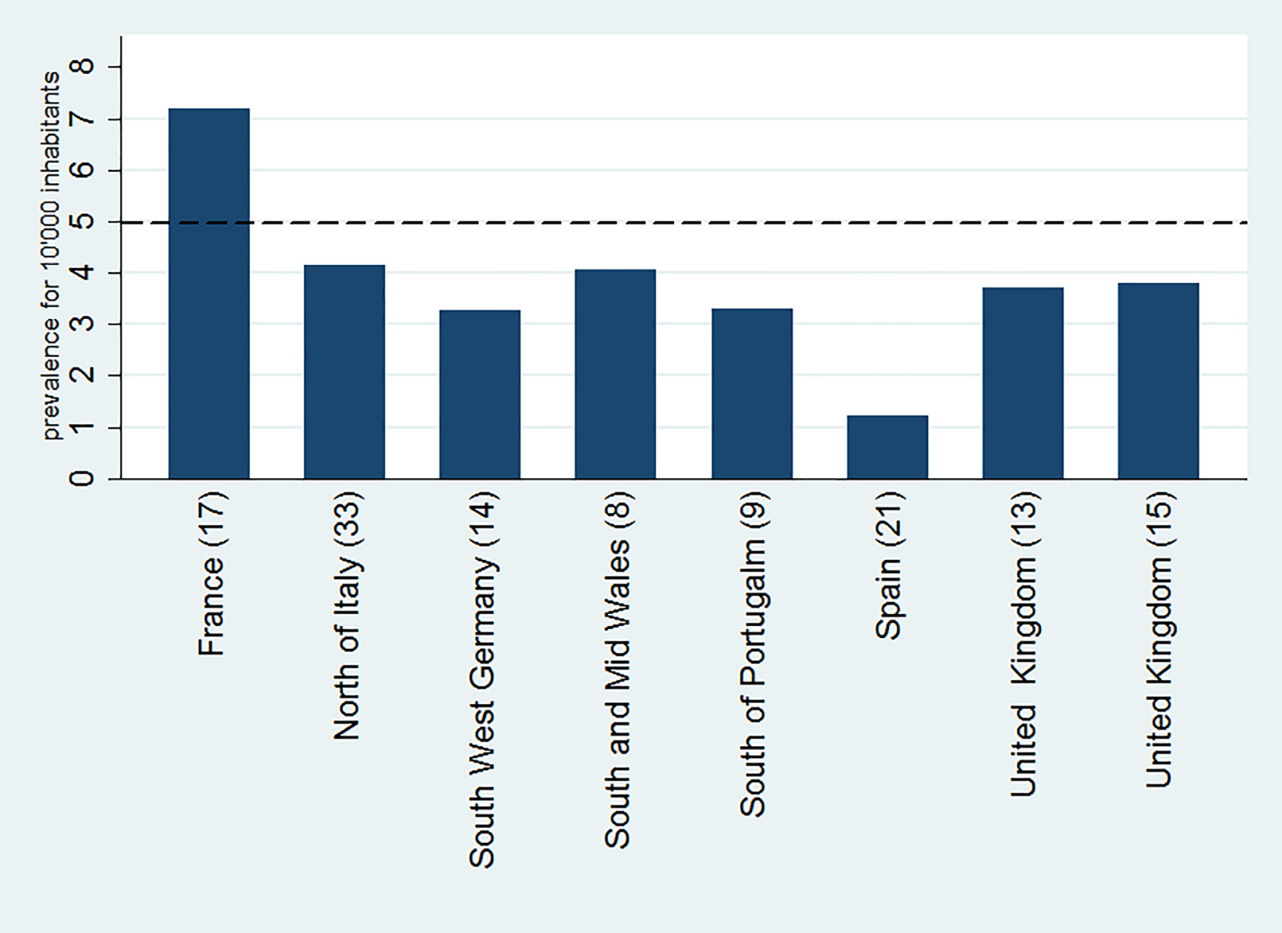
The figure depicts the predicted prevalence reported in the selected epidemiological studies[9, 10, 14–16, 18, 22, 35]. Point prevalence was reported if predicted prevalence was not available. the dotted line indicates the limit of 5 cases: 10,000 inhabitants adopted by EMA to define rare disease.

**Fig 3:**
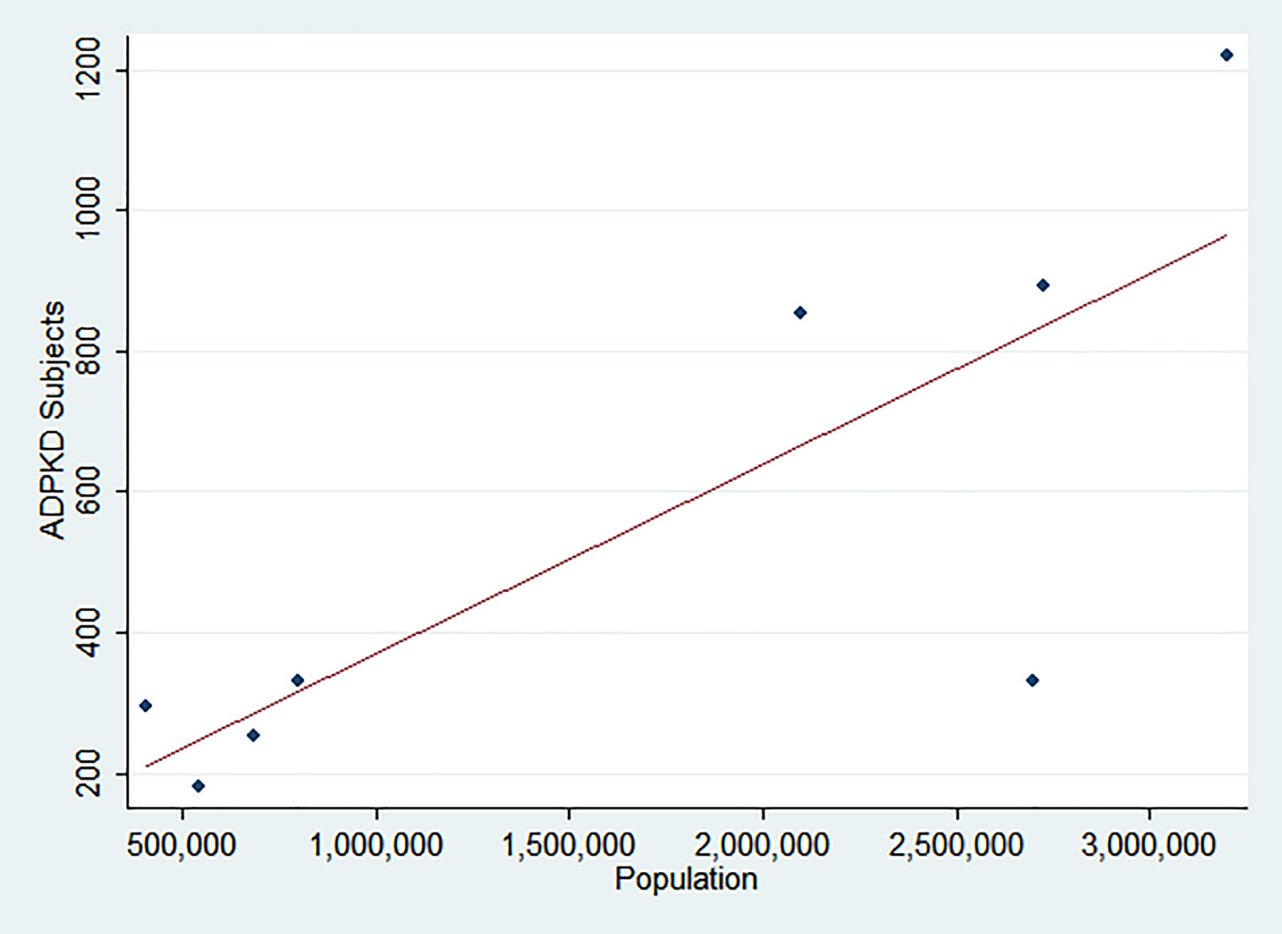
Plot of the ADPKD subjects over the reference population reported in the selected articles ([9, 10, 14–16, 18, 22, 35]). Dashed line represents linear regression of the plotted points.

Table 1 summarizes the point prevalence and predicted prevalence that is either directly reported or indirectly obtainable from the data of these selected publications. ‘Predicted Prevalence’ represents the effort of the authors to handle missing diagnoses using heterogeneous methodological approaches. These data are available in two analyzed articles [9, 10]. Where not available, the point prevalence has been used in the following analysis. The same data are represented in Fig 2. We performed a linear regression of the ADPKD population over the reference population reported in the articles. Fig 3 plots the estimations of the reported prevalences. After linear regression, the combined prevalence of these studies is 2.7: 10,000 subjects (CI 95: 0.73–4.67: 10,000 subjects). The Cochran’s Q suggests heterogeneity between studies (Q = 184.78; p<0.001).

**Table 1:** Selected epidemiological studies.

Reference	[9]	[10]	[15]	[16]	[18]	[14]	[35]	[22]
Point Prevalence	1.44: 10,000	1.54: 10,000	3.27: 10,000	3.81: 10,000	7.2: 10,000	3.72: 10,000	4.16: 10,000	1.23: 10,000
Predicted Prevalence	4.06: 10,000	3.31: 10,000	NA	NA	NA	NA	NA	NA
Geographic Region	South and Mid Wales	South of Portugal	South West Germany	United Kingdom	France	United Kingdom	North of Italy	Spain

Point prevalence, predicted prevalence and geographic region of the selected epidemiological studies[9, 10, 14–16, 18, 22, 35]

### Point prevalence of ADPKD in the province of Modena

According to the data sources described in the methods section, we identified 238 index cases belonging to 184 different families. We made an attempt to contact each of these subjects and invited them for a visit in our center. All the patients that accepted to be enrolled in the study and to be interviewed signed the consent form. The clinical evaluation of the compliant subjects (141 individuals, 59.3%) allowed us to collect 99 pedigrees; however, in the remaining cases it was neither possible to contact the subject nor extract an address or phone number of 66 subjects from the source (27.7%); also, 21 subjects did not reply to phone or mail invitation to the study (8.8%). Ten subjects have not been compliant to a clinical visit in our center (4.2%). This initial data collection and pedigree analysis generated a list of 493 subjects reported as either affected (238 subjects) or at-risk subject of ADPKD (255 subjects) (see **Table 2**).

**Table 2:** Collection of diagnostic data of at risk subjects in the province of Modena.

						Clinically Defined
**184 families**	Affected (index cases)	238				238
	At Risk (99 pedigrees)	255	With Exams: 86	Affected (Post Test)	16^■^	16
				Undefined (Post Test)	21^■^	
				Not Affected (Post Test)	43^■^+6^▲^	
			Without Exams: 169 (Undefined Pre Test)			
**TOTAL**						254

Summary of 493 subjects collected during the survey. In 85 patients in the at-risk group, diagnostic tests (imaging exam■ and/or genetic test▲) were available.

After completion of the clinical visits, 16 at-risk subjects resulted in being actually affected according to imaging (US or MRI diagnosis). In 43 at-risk subjects, the diagnosis was excluded by imaging and six subjects were excluded on the basis of a genetic test. Twenty-one at-risk subjects did not reveal any conclusive imaging or genetic tests. After the clinical evaluation of the compliant subjects, affected patients totaled 254. According to the National Institute of Statistical (ISTAT, 2016), the population of the Province of Modena, comprises 701,642 inhabitants. According to a more conservative calculation considering only the clinically defined affected patients, the point prevalence can be reported as 3.63: 10,000 (CI 95% = 3.010–3.758). This estimate shows the prevalence of clinically diagnosed and probably underestimates the real prevalence because of a number of underdiagnosed subjects. Considering that patients with stage 1 and 2 of renal failure were more likely to be underdetected, we performed a sensitivity analysis, assuming percentages of subjects in stage 1–2 of 40%, 50% and 60%. In these three scenarios, the prevalence varies from a minimum of 3.97 to a maximum of 5.96: 10,000 inhabitants (see Table J in S1 File in supplemental material for details)

### Predicted prevalence of ADPKD in the province of Modena

**Fig 4:**
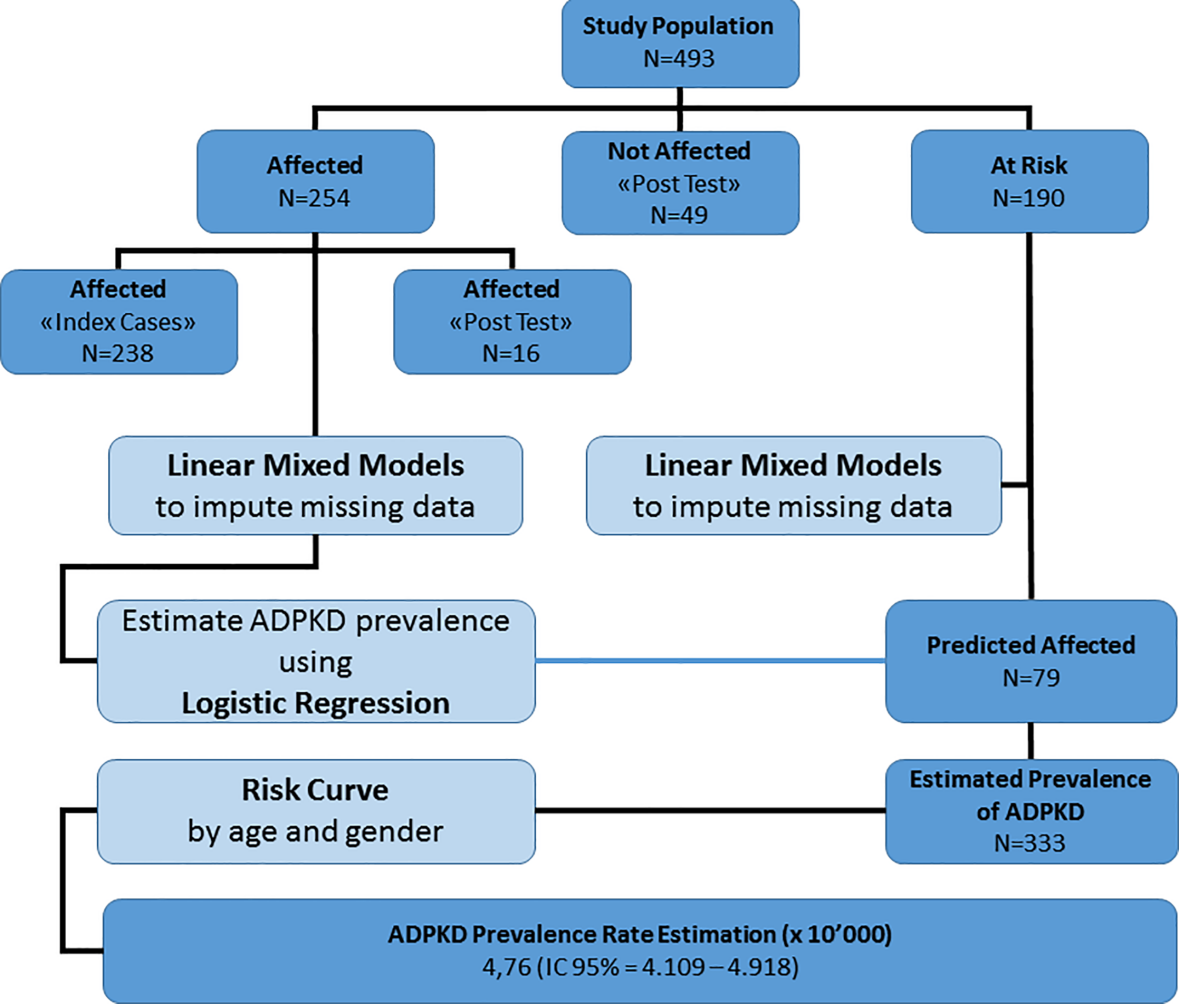
Flow chart of the statistical approach for the calculation of the predicted prevalence of ADPKD.

The strategy adopted for the estimation of the Predicted Prevalence of our population is summarized in Fig 4. The process preliminarily required the imputation of missing data (age, gender, Family Risk Score; see Materials and Methods for a definition and calculation of the Family Risk Score) using a linear mixed model both in the clinically-defined affected subjects and in the at-risk population. Finally, a logistic regression model based on a set of predictor variables (age, gender, Family Risk Score) was used to predict the outcome (affected status) in the at-risk population.

Finally, the age-adjusted prevalence rate was defined by considering the age distribution of the population of the Province of Modena, according to ISTAT survey performed in January 2016.

Applying the Logistic Regression model, we calculated the expected number of affected patients in the at-risk population that had not participated in an instrumental exam (pretest population). Based our findings, we noted that 79 of 190 (41.6%) subjects would be affected in the at-risk population. The overall number of predicted affected patients (254+79 subjects) resulted in 333 estimated ADPKD subjects. Accordingly, the Predicted Prevalence can be estimated in 4.76: 10,000 inhabitants (CI 95% = 4.109–4.918).

The Family Risk Score incorporates the rate of subjects without a family history that in our population is 15.1% and it is approximated to 15% in the model. Furthermore we have adjusted this value from 10% to 15% and to 20% in a sensitive analysis to verify the impact of this factor in our prediction (see **Table 3**).

**Table 3:** Sensitivity analysis of predicted prevalence according to variation of the percentage of ADPKD subjects without family history.

*PERCENTAGE OF SUBJECTS WITHOUT FAMILY HISTORY*	*PREDICTED PREVALENCE IN 10*,*000 INHABITANTS*	*CI 95% OF PREDICTED PREVALENCE*
**10%**	4.79	4.123–4.945
**15%**	4.76	4.109–4.918
**20%**	4.72	4.074–4.877

### Characteristics of the cohort

The compliant patients that accepted a complete clinical evaluation numbered 141 (55.5%) subjects out of 254 identified ADPKD subjects. **Table 4** reports the main characteristics of this population. A positive family history of ADPKD is present in 120 subjects (84.9%). We found that the male gender is slightly more frequent, with a median age of the cohort being 56.1 years. Forty-three patients (30.5%) were in renal replacement therapy, with transplantion being the most prevalent approach: 20 patients (46.5%). The median age of starting renal replacement therapy is 54 years (25°-75° 47–62 years). Gender is neither significantly different in the male/female ratio of subjects in RRT (16 females, 27 males; p 0.196), nor in the age of starting RRT (female median 55 years [25°-75° 49–61.5 years]; male median 52 years [25°-75° 42–62 years]; p 0.529), although both parameters are tendentially worse in subjects of male gender.

**Table 4:** The major clinical characteristics of the cohort.

*M:F*	1.085: 1	
*Age (years)*	58.3 (45.9–66.7)	
*CKD Class:*		
*1*	21 (15%)	
*2*	27 (19.2%)	
*3a*	15 (10.8%)	
*3b*	11 (7.5%)	
*4*	14 (10%)	
*5*	53 (37.5%)	*43 of 53 requiring RRT:* - *Hemodialysis: 18* (*41.9%)* - *Peritoneal Dialysis: 5* *(11.6%)* - *Transplant: 20* *(46.5%)*
Presence of Hypertension	119 (84.7%)	
Age of Hypertensive state onset (years)	39 (30–49)	
Total Kidney Volume (ml) *	1641.83 (885.57–2565)	
Mayo Clinic Score*:		
*1A*	1 (2%)	
*1B*	14 (28%)	
*1C*	16 (32%)	
*1D*	14 (28%)	
*1E*	5 (10%)	
*PROPKD Score^▲^:*		
*0–3*	19 (53.6%)	
*4–6*	12 (32.1%)	
*7–9*	5 (14.3%)	

Data are expressed as Median (25°-75°) or number of subjects(%).

* Data of 50 patients were available for Total Kidney Volume.

▲ The data of 36 subjects were available for the PKDSCORE.

Hypertension is a common complication that is reported in 119 patients (84.7%). The median age of onset of this condition is 39 years. Hypertension is slightly less common in females (80.6%) than males (88%); however, age of onset is not statistically different between genders (p 0.693). Kidney Volume was available for 50 patients included in this study. According to the Mayo Clinic ADPKD score [36], patients in class 1B, 1C, and 1D each represent approximately 30% of the sample, while patients in class 1A and 1E are less common (see **Table 4**).

Twelve cases indicated a positive family history of cerebral aneurysm, while two more subjects reported a positive family history for subarachnoid hemorrhage. Five subjects reported the presence of aneurysms (none of them had a positive family history for intracranial aneurysms). One subject reported a previous subarachnoid hemorrhage (without a positive family history). Forty-one subjects reported a negative MRI cerebral scan, while the remaining cases never underwent a neuroradiologic examination having a negative family history.

### Genetic analysis

Genetic analysis for PKD1 and PKD2 were available on a subset (42 subjects) of at-risk or affected subjects in our cohort (**Table 5**). In 23 patients, we performed the complete analysis of both genes using Sanger sequencing, while in 19 related patients, we evaluated the segregation analysis of the previously-identified pathogenic variant of the family. Six of these 19 patients resulted negative at molecular analysis and were classified as not affected.

**Table 5:** Table of genetic variants identified in the cohort.

Patient ID	Sequencing	Variant (c.DNA)	Exon/intron	Protein	Type of mutation		Functional domain	PKDB database	ACMG
**PKD1**									
**1**	complete	c.393_394del TG	4	p.Cys131Trpfs*47	T	frameshift		Definitely Pathogenic	Pathogenic
**2**	complete	c.2639_2649del11	11	p.Thr880_Pro883delinsArgfs*21	T	frameshift		Definitely Pathogenic	Pathogenic
**3**	complete	c.2884delG	12	p.Asp962Thrfs*14	T	frameshift	PKD domain		Pathogenic
**4**	complete	c.9240_9241delAT	26	p.Ala3082Cysfs*96	T	frameshift	Polycystic kidney disease type 1 protein		Pathogenic
**5**	complete	c.9996delT	30	p.Val3332fs*63	T	frameshift	Polycystic kidney disease type 1 protein		Pathogenic
**6**	complete	c.4551C>A	15	p.Tyr1517*	T	nonsense	PKD domain	Definitely Pathogenic	Pathogenic
**7**	complete	c.5477G>A	15	p.Trp1826*	T	nonsense	PKD domain	Definitely Pathogenic	Pathogenic
**8**	complete	c.8095C>T	22	p.Gln2699*	T	nonsense	REJ domain		Pathogenic
**9**	complete	c.9559_9561delGAC	27	p.Asp3187del	IF	in frame	PLAT/LH2 domain		Likely pathogenic
**10**	segregation	c.9559_9561delGAC	27	p.Asp3187del	IF	in frame	PLAT/LH2 domain		Likely pathogenic
**11**	complete	c.11270-3C>A	39i		T	atypical splicing			Likely pathogenic
**12**	complete	c.9397+169C>G	5i		NT	atypical splicing		Likely Pathogenic	Uncertain significance (VUS)
**13**	complete	c.12444+57_81del	45i		T	intronic			Likely pathogenic
**14**	complete	c.194T>A	1	p.Ile65Asn	NT	missense			Likely benign
**15**	complete	c.6137T>C	15	p.Leu2046Pro	NT	missense	PKD/Chitinase domain		Likely pathogenic
**16**	segregation	c.6137T>C	15	p.Leu2046Pro	NT	missense	PKD/Chitinase domain		Likely pathogenic
**17**	segregation	c.6137T>C	15	p.Leu2046Pro	NT	missense	PKD/Chitinase domain		Likely pathogenic
**18**	complete	c.6749C>T	15	p.Thr2250Met	NT	missense	PKD/Chitinase domain	Likely Neutral	Uncertain significance (VUS)
**19**	complete	c.7300C>T	18	p.Arg2434Trp	NT	missense	PKD/REJ-like domain	Highly Likely Pathogenic	Likely pathogenic
**20**	segregation	c.7300C>T	18	p.Arg2434Trp	NT	missense	PKD/REJ-like domain	Highly Likely Pathogenic	Likely pathogenic
**21**	complete	c.9499A>T	27	p.Ile3167Phe	NT	missense	PLAT/LH2 domain	Indeterminate	Likely pathogenic
**22**	segregation	c.9499A>T	27	p.Ile3167Phe	NT	missense	PLAT/LH2 domain	Indeterminate	Likely pathogenic
**23**	segregation	c.9499A>T	27	p.Ile3167Phe	NT	missense	PLAT/LH2 domain	Indeterminate	Likely pathogenic
**24**	segregation	c.9499A>T	27	p.Ile3167Phe	NT	missense	PLAT/LH2 domain	Indeterminate	Likely pathogenic
**25**	complete	c.11537+2T>A	41i		T	splicing			Pathogenic
**26**	segregation	c.6548c>t	15	p.Thr2183Ile	NT	missense			Likely pathogenic
**27**	segregation	c.6548c>t	15	p.Thr2183Ile	NT	missense			Likely pathogenic
**28**	complete	c.12061C>T	44	p.Arg4021*	T	nonsense		Definitely Pathogenic	Pathogenic
**29**	complete	c.6307C>T	15	p.Gln2103*	T	nonsense		Definitely Pathogenic	Pathogenic
**30**	complete	c.8364G>A	23	p.Ser2788Ser	NT	synonymous	REJ domain	Likely Neutral	Uncertain significance (VUS)
**PKD2**									
**31**	segregation	c.1158T>G	5	p.Tyr386*	T	nonsense	Polycystin cation channel, PKD1/PKD2		Pathogenic
**32**	segregation	c.2614C>T	14	p.Arg872*	T	nonsense			Pathogenic
**33**	segregation	c.2614C>T	14	p.Arg872*	T	nonsense			Pathogenic
**34**	complete	c.843+1G>T	3i		T	splicing		Definitely Pathogenic	Pathogenic
**35**	segregation	c.843+1G>T	3i		T	splicing		Definitely Pathogenic	Pathogenic
**36**	complete	c.1094+1G>A	4i		T	splicing		Definitely Pathogenic	Pathogenic

The PKDB database (Mayo Clinic) column reports the variant pathogenicity available at ‘http://pkdb.mayo.edu/’. ACMG column reports the pathogenicity classification performed by our group according to Richards et al.[25]. Moecular analysis was performed in 42 subjects, 6 subjects with a negative segregational result are not reported.

**Fig 5:**
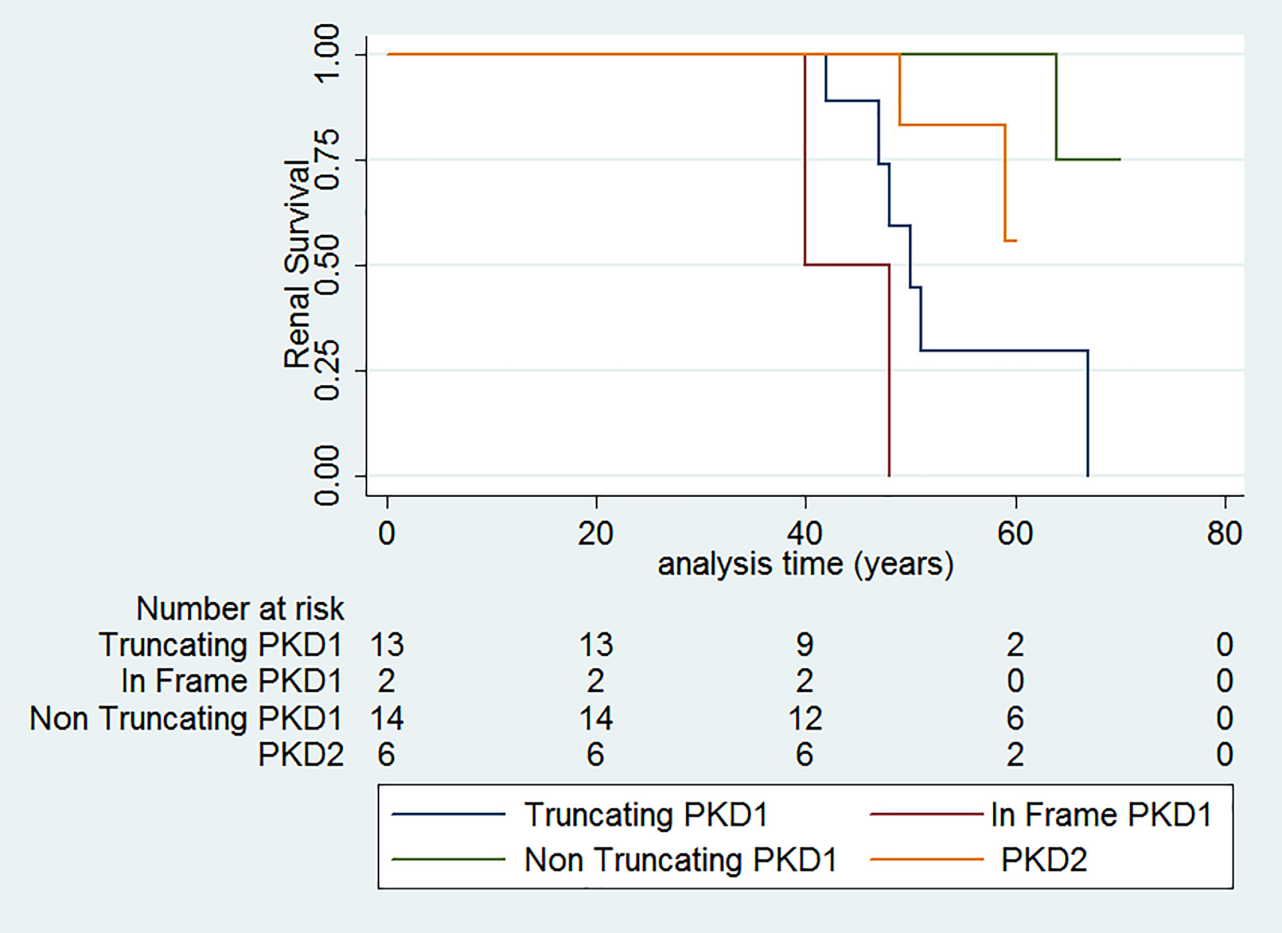
Renal survival of patients according to the type of their variant.

Characteristics of the variants for each of the genotyped subjects are reported in Table 5. The distribution of the PROPKD score [30] is reported in **Table 4**. Renal survival is modified by mutation classes (p<0.001) (the number of truncating and not truncating variants are reported in Table K in S1 File of the supplemental material, while the survival curves are represented in Fig 5.

## Discussion

This work is, to our knowledge, the first attempt to produce a meta-analytical synthesis of the epidemiological literature available in Europe on ADPKD prevalence in the general population. Furthermore the estimation of missing data (predicted prevalence) has been obtained in our cohort by an unprecedented strategy (logistic regression prediction). ADPKD epidemiology is a controversial topic with highly variable estimates of prevalence in the available literature, ranging from 1.44: 10,000 [9] to 25: 10,000 [4]. Several reasons may concur this variability. The major factor that introduces uncertainty in estimates is the presence of a significant proportion of affected but asymptomatic patients (patients that will become clinically evident later in their life) and the lack of a simple and inexpensive population screening tool. Other components of the analysis may introduce variability in the epidemiology of ADPKD; for example, the nature of the sources used in patient research, and the epidemiological and statistical techniques used to remedy missing data, etc. Finally, the regional variability of the incidence of the disease can not be excluded, indeed at least the founder effect has been documented in small and isolated communities [21].

Hospital databases and the collection of data from clinical notes, which are frequently adopted as epidemiological source material, necessarily introduce the bias of missing asymptomatic ADPKD subjects. A population-wide imaging-based screening for ADPKD has never been performed and it will probably never be done in consideration of the high costs. The prevalence estimates reported target clinical cases mostly likely presented to nephrologists/internists. Many patients with good kidney function cared by primary care physicians might not be captured in the published literature and the same underestimation is highly probable in the selected studies of our meta-analysis. However, in the epidemiological study of the province of Modena, we have attempted to reduce this bias both in the point and in the predicted estimation by two different strategies: the detailed reconstruction of the pedigrees of our index cases and the prediction of affected cases by a logistic regression model. Logistic regression is a statistical tool useful to predict the presence or absence of an outcome (ADPKD) based on values of a set of predictor variables (age, gender, Family Risk Score). It is similar to a linear regression model but is suited to models where the dependent variable is dichotomous (presence/absence of ADPKD). Gender and age are well recognized modifying variables of ADPKD and confirmed by our epidemiological study. ADPKD has a mild male prevalence among affected subjects, whereas ADPKD is most likely to be diagnosed in adult subjects compared to pediatric subjects.

The Family Risk Score was defined as the ratio of affected subjects compared to those at risk in a family. The predicting value of this parameter relies on the well documented role of genetic variant in the severity of the condition. The same genetic variant, and its disease risk, is shared by all the affected components of the same family. The value of the covariate “Family Risk Score” is included in the logistic model and adjusted for possible cases without an apparent family history (15% of cases).

The first part of our work consisted in a meta-analysis of epidemiological data of the literature. An extensive bibliographic research identified eight relevant articles. A synthetic analysis of data extracted from this source allowed us to establish a prevalence of disease of 2.7: 10,000 subjects (CI 95: 0.73–4.67: 10,000 subjects). Although the meta-analytic approach permits to evaluate a significant European reference population (over 13,000,000 subjects), the intrinsic limit of this analysis is the heterogeneity of the epidemiological approaches used to estimate the prevalence by the different authors.

To overcome the uncertainty of the estimate originating from a meta-analytic elaboration, in the second part of our work we directly investigated the prevalence of the condition in our region. One of the strengths of the epidemiological approach to this work has been the effort of maximizing the collection of diagnosed subjects, mainly based on pedigree collection and extensive database search; nevertheless, our approach may have suffered from bias. In particular, despite our attempt to contact each affected and at-risk subject, we still managed to clinically evaluate a proportion of our cohort faraway from completeness, limited compliance being an inherent weakness in this type of study. In consideration of the risk of missing diagnosis, our point prevalence (3.63: 10,000) most likely represents an underestimation of the real prevalence of the condition. Our point prevalence is in line with the average estimates of the majority of the previous epidemiological studies[9, 10, 14–16], with only two studies reporting higher prevalence[18, 35]. In the attempt of correcting for the missing diagnosis, we calculated an estimated prevalence by applying a prediction of affected subjects in the clinically uncharacterized at-risk population. Our estimated prevalence of ADPKD (4.76:10’000) represents the highest estimation of all the previous epidemiological studies, with the only exception of the French study [18]. A recent paper [37] compared population-based and renal registry studies and concluded that, under specific assumptions (inflation rate, etc.), data are consistent between the two approaches. They proposed an estimated prevalence of 3.96:10,000, a figure close, but still lower than our predicted estimation. Interestingly, a public document produced by the Committee for Orphan Medicinal Products (COMP—Minutes of the 16–18 June 2015 meeting) on the orphan drug designation of Lanreotide, a somatostatin analog for treatment in ADPKD, reported a prevalence of between 4.2 and 4.7 in 10’000. Even if this document does not provide details about the adopted epidemiological methods, it reports an interval that is higher than the prevalent previous literature and particularly close to our confidence limits (4.109 and 4.918 in 10’000). The significant divergence between point prevalence and predicted prevalence, as described in our study, suggests that there is a significant proportion of unrecognized patients. In our analysis, these subjects could account for about 25% of the overall ADPKD population in our province. The fact that these patients did not come to clinical attention does not mean that they would not deserve it. In fact, we can suspect that the presence of early and not symptomatic complications, such as hypertension or eligibility to specific treatments [38] could be not uncommon in this category of undiagnosed subjects. However, it is reasonable to predict that a proportion of these underdiagnosed subjects may have a milder clinical picture compared to those already captured in clinical files.

The efforts made in this study to identify all affected patients in a defined geographical area hopefully produced a more significant picture of the distribution of relevant clinical data compared to some of the previous descriptions based on potentially biased collection (e.g., dialytic populations, imaging archives, genetic registers, etc.). We have identified a relatively high frequency of subjects without a family history of ADPKD (15.1%). This data is usually rarely reported in the literature; for example, in the selected epidemiologic studies, [9, 10, 14–16, 18, 35] only 3 [9, 18, 35] report it in a range from 1.6% to 16%. One recent population-based study has reported about 15% of cases with confirmed de novo mutation and another 10% without an apparent family history [39]. In our study, the rate of family history of ADPKD was close to the percentage of the HALT- PKD reports [40, 41]. Hypertension is the first complication for age of onset and frequency in ADPKD. We found an elevated frequency (84.7%) of this complication in our cohort, which was higher than previously reported (50–80%)[16, 42, 43], but with a later median age of onset (9 years) than reported by Schrier et al.[44].

In our cohort, six subjects had intracranial aneurysms (one of them had a previous aneurysmatic rupture) that represent a 4.2% of presence of this complication. Notably, none of these subjects reported a positive family history for intracranial aneurysms or subarachnoid hemorrhage.

In conclusion, we conducted an epidemiological study that sought to maximize the identification of the affected subjects using an in-depth pedigree analysis. We also adopted an unprecedented strategy to adjust our estimation of prevalence with respect to possible missing diagnoses in at-risk subjects. The Point Prevalence (3.63:10,000) of our study is in line with the average of the literature estimates. In contrast, predicted prevalence (4.76:10’000) indicates a generally higher rate of disease than previously reported and suggests a significant proportion of missed diagnoses in at-risk patients. This could be attributed to the low attention given by nephrologists to family history and the reconstruction of the genealogical tree in outpatient activity, which, on the contrary, has been extensively applied in our study. Nevertheless, our prevalence estimates, which do not exceed the limit of 5:10,000 inhabitants, are still compatible with the definition of rare disease adopted by the European Medicines Agency and Food and Drug Administration.

## Supporting information

S1 File Supporting tables and figures. (DOCX)

S2 File Minimal manuscript dataset of the prevalence study. (XLSX)

S3 File Minimal manuscript dataset of the clinical description of the cohort. (XLSX)
