# Supplementary material for: The prevalence of autosomal dominant polycystic kidney disease (ADPKD): A meta-analysis of European literature and prevalence evaluation in the Italian province of Modena suggest that ADPKD is a rare and underdiagnosed condition
Source: PLoS One. 2018 Jan 16;13(1):e0190430. doi: 10.1371/journal.pone.0190430 (PMC5770025; doi:10.1371/journal.pone.0190430)
Supplement: S1 File — (DOCX) [file pone.0190430.s001.docx]

Supplemental Materials

## Title : ‘The prevalence of Autosomal Dominant Polycystic Kidney Disease (ADPKD): a meta-analysis of European literature and prevalence evaluation in the Italian province of Modena suggest that ADPKD is a rare and underdiagnosed condition’.

Authors : Andrea Solazzo^1¶^, Francesca Testa^1¶^, Silvia Giovanella^1^, Marco Busutti^2^, Luciana Furci^3^, Paola Carrera^4^, Maurizio Ferrari^4,5^, Giulia Ligabue^1^, Giacomo Mori^3^, Marco Leonelli^3^, Gianni Cappelli^1,3^, Riccardo Magistroni^1,3*^

**Appendix A. Imaging criteria for diagnosis of ADPKD**

**Appendix B. Demographic, Clinical and Molecular data collected during the study**

**Appendix C. Methods of molecular genetic characterization**

**Tables A-H. Synthetic characteristics of selected epidemiologic studies**

**Table I.** **Newcastle-Ottawa Quality Assessment table**

**Table J. Sensitivity analysis of ADPKD prevalence assuming different distribution of CKD stages in the population.**

**Table K. Type of variants (truncating or not truncating) of our cohort**

**Table L. Tables of logistic regression analysis.**

**Table M. Curve estimation cubic model summary and parameters results.**

**Fig A. The distribution of the cumulative risk to be affected according to age and differentiated by sex** .

**Fig B. Distribution of the Family Risk Score (affected/at risk subjects) in 91 families collected in the study.**

**Appendix A. Imaging criteria for diagnosis of ADPKD**

Ultrasound

The diagnostic criteria for ADPKD were based on imaging evaluation by ultrasound according to Pei et al. [23] :

- age 15-29 years equal or greater than 3 cysts unilaterally or bilaterally [PPV 100% VPN 85.5%]
- age 30-39 years equal or greater than 3 cysts unilaterally or bilaterally [PPV 100% and 96.4% VPN]
- age 40-59 years equal or greater than 2 cysts for each kidney [VPP 100% and 94.8% VPN]).

Subjects without cysts and age older than 40 years have been considered not affected.

Magnetic Resonance Image (MRI)

MRI has been considered a valid exam for diagnosis. We adopted the diagnostic criteria reported by Pei et al. [24]:

- people aged between 16-40 years with a number > 10 cysts have been considered affected;
- subjects aged 16-40 years with less than < 5 cysts have been considered not affected).

**Appendix B. Demographic , Clinical and Molecular data collected during the study**

The data collected included patient demographics, molecular and clinical informations: age, gender, Family number, ID number, family history, age of first diagnosis, replacement therapy (hemodialysis, peritoneal dialysis, kidney transplantation), hypertension, diabetes, urological complications, cerebrovascular accidents (Intracranial aneurysm, stroke, intraparenchimal haemorrage, subarachnoid haemorrage), height, weight, blood pressure, heart beat rate, therapy, genetic variants, prognostic score ( PROPKD Score [30], MAYO score [36]).

**Appendix C. Methods of molecular genetic characterization**

In brief, amplification of the coding region of PKD1, avoiding the pseudo-genes, was obtained by 8 Long Range and 43 nested PCR . This strategy amplified exons 1–33 and exon-intron junctions. Exons 34 to 46 of PKD1 as well as the all exons of PKD2 were amplified from genomic DNA in a single PCR.

In patients with no variants detected by Sanger sequencing, a MLPA (Multiple Ligation Probe Assay) analysis has been performed.

For the position's evaluation we have used the reference sequences: ENST00000262304, ENSG00000008710 for PKD1 and ENST00000237596, ENSG00000118762 for PKD2.

For each variants we have defined the nucleotide position, in according to HGVS nomenclature (Human Genome Variation Society), aminoacid position, functional domain and type of mutation. Type of variants are reported in Supplemental Table 8. The frequency of variants has been evaluated in public databases (Exac, 1000 genomes and Exome Variant Service), and bioinformatics algorithms have been adopted for the prediction of functionality impact of variant (Mutation Taster, GERP, A-GVGD, PolyPhen-2 and SIFT). When it was advisable ^21^ and family members were compliant we have performed segregational analysis. The variants were classified according to ACMG guidelines (“Pathogenic”, “Likely pathogenic”, “Uncertain significance”, “Likely benign” and “Benign”) [25].

**Tables A-H. Synthetic characteristics of selected epidemiology studies**

**Table A**

| (No.) Reference | [9] |
| --- | --- |
| Type of Source | Original Article |
| Region | South and Mid Wales |
| Collection Year(s) | 1982-1989 |
| Case definition | ADPKD diagnosis by ultrasound : ‘if both kidneys contained cysts and at least one contained two or or more cysts’ |
| Data Collection Method | Pedigree collection from index cases identified in the RRT population. Patients attending renal clinics. Request to physicians and nephrologists of the region to notify patients |
| Design | Genetic registry of all known cases |
| Reference Population | South and Mid Wales population (2.1 million people served by 7 nephrologists in 4 centers) |
| Other Incidence (I)  Renal Replacement Therapy (RRT)  Mortality (M) | 1. NA   (RRT) 4.8 : 1.000.000  (M) NA |
| Calculated prevalence | Point Prevalence : 1.44 : 10.000  Predicted Prevalence : 4.06 : 10.000 |
| Newcastle-Ottawa Quality Assessment | Selection Stars 2  Comparability Stars 0  Outcome Stars 1  AHRQ standard Poor quality |
| Comments | Predicted Prevalence has been calculated for patients at risk and with age younger than 30 years |

**Table B**

| (No.) Reference | [10] |
| --- | --- |
| Type of Source | Letter to Editor |
| Region | Alentejo (South of Portugal) |
| Collection Year(s) | (1999 – 2001 ) |
| Case definition | Not Specified |
| Data Collection Method | Pedigree collection from index cases identified in the RRT population. Patients attending renal clinics. |
| Design | Registry of all known cases |
| Reference Population | 543.442 inhabitants |
| Other Incidence (I)  Renal Replacement Therapy (RRT)  Mortality (M) | (I) NA  (RRT) NA  (M) NA |
| Calculated prevalence : Point Prevalence (PoP)  Predicted Prevalence (PrP) | PoP : 1.54 : 10.000  PrP : 3.31 : 10.000 |
| Newcastle-Ottawa Quality Assessment | Selection Stars 2  Comparability Stars 0  Outcome Stars 1  AHRQ standard Poor quality |
| Comments | PrP has been calculated with a fixed genetic risk of 50% for at risk subjects without any age adjustment. |

**Table C**

| (No.) Reference | [15] |
| --- | --- |
| Type of Source | Original Article |
| Region | South West Germany |
| Collection Year(s) | 2004-2010 |
| Case definition | ‘internationally approved standard criteria’ (ultrasound criteria) |
| Data Collection Method | Survey by a questionaire |
| Design | Registry (Else-Kroener-Fresenius-Registry of ADPKD) |
| Reference Population | 2.727.351 inhabitants |
| Other Incidence (I)  Renal Replacement Therapy (RRT)  Mortality (M) | (I) NA  (RRT) 10.4 : 1000.000  (M) NA |
| Calculated prevalence : Point Prevalence (PoP)  Predicted Prevalence (PrP) | PoP : 3.27 : 10.000  PrP : NA |
| Newcastle-Ottawa Quality Assessment | Selection Stars 3  Comparability Stars 0  Outcome Stars 1  AHRQ standard Poor quality |
| Comments |  |

**Table D**

| (No.) Reference | [16] |
| --- | --- |
| Type of Source | Original Article |
| Region | United Kingdom |
| Collection Year(s) | 1991-2008 |
| Case definition | Recorded diagnostic code : ‘Polycystyc Kidney Disease’, ‘Polycystic Kidney’ , ‘Polycystic Disease Kidney’ |
| Data Collection Method | Search for ADPKD patients from the UK General Practice Research Database older than 15 years |
| Design | Registry Cohort Study |
| Reference Population | 3.2 million |
| Other Incidence (I)  Renal Replacement Therapy (RRT)  Mortality (M) | (I) NA  (RRT) 32.5 : 1000.000  (M) NA |
| Calculated prevalence : Point Prevalence (PoP)  Predicted Prevalence (PrP) | PoP : 3.81 : 10.000  PrP : NA |
| Newcastle-Ottawa Quality Assessment | Selection Stars 2  Comparability Stars 0  Outcome Stars 1  AHRQ standard Poor quality |
| Comments |  |

**Table E**

| (No.) Reference | [18] |
| --- | --- |
| Type of Source | Original Article |
| Region | France |
| Collection Year(s) | 1988-1993 |
| Case definition | NA |
| Data Collection Method | NA |
| Design | Retrospective Cohort Study |
| Reference Population | 410.000 |
| Other Incidence (I)  Renal Replacement Therapy (RRT)  Mortality (M) | (I) NA  (RRT) 32.5 : 1000.000  (M) NA |
| Calculated prevalence : Point Prevalence (PoP)  Predicted Prevalence (PrP) | PoP : 7.2 : 10000  PrP : NA |
| Newcastle-Ottawa Quality Assessment | Selection Stars 2  Comparability Stars 0  Outcome Stars 1  AHRQ standard Poor quality |
| Comments | Conflicting data in presented results 296 affected subjs; 410000 inhabitants (7.2 : 10000); reported prevalence 1:1111 (9 : 10000) |

**Table F**

| (No.) Reference | [14] |
| --- | --- |
| Type of Source | Original Article |
| Region | United Kingdom |
| Collection Year(s) | 2006-2010 |
| Case definition | Clinical code for polycystic kidney disease |
| Data Collection Method | Cross sectional analysis on people included in Quality Improvement in Chronic Kidney Disease (QICKD) trial database |
| Design | Retrospective Cohort Study |
| Reference Population | 684.512 |
| Other Incidence (I)  Renal Replacement Therapy (RRT)  Mortality (M) | (I) NA  (RRT) NA  (M) NA |
| Calculated prevalence : Point Prevalence (PoP)  Predicted Prevalence (PrP) | PoP : 3.72 : 10000  PrP : NA |
| Newcastle-Ottawa Quality Assessment | Selection Stars 3  Comparability Stars 0  Outcome Stars 2  AHRQ standard Poor quality |
| Comments | Excluded subjects older than 60 years |

**Table G**

| (No.) Reference | [35] |
| --- | --- |
| Type of Source | Original Article |
| Region | North of Italy |
| Collection Year(s) | 2007-2009 |
| Case definition | Ultrasound Criteria based on Ravine et al. and Pei et al. (20, 40) |
| Data Collection Method | Collection of patients referred to the Department of Vicenza |
| Design | Retrospective Cohort Study |
| Reference Population | 800.000 |
| Risk Factors |  |
| Other Incidence (I)  Renal Replacement Therapy (RRT)  Mortality (M) | (I) NA  (RRT) NA  (M) NA |
| Calculated prevalence : Point Prevalence (PoP)  Predicted Prevalence (PrP) | PoP : 4.16 : 10000  PrP : NA |
| Newcastle-Ottawa Quality Assessment | Selection Stars 3  Comparability Stars 0  Outcome Stars 1  AHRQ standard Poor quality |
| Comments |  |

**Table H**

| (No.) Reference | [22] |
| --- | --- |
| Type of Source | Original Article |
| Region | Spain |
| Collection Year(s) | 1991 |
| Case definition | Ultrasound (not better defined) |
| Data Collection Method | Questionnaire sent to nephrological centers |
| Design | Retrospective Cohort Study |
| Reference Population | 2.700.000 |
| Risk Factors |  |
| Other Incidence (I)  Renal Replacement Therapy (RRT)  Mortality (M) | (I) NA  (RRT) 0.75 : 10’000  (M) NA |
| Calculated prevalence : Point Prevalence (PoP)  Predicted Prevalence (PrP) | PoP : 1.23 : 10’000  PrP : NA |
| Newcastle-Ottawa Quality Assessment | Selection Stars 2  Comparability Stars 0  Outcome Stars 1  AHRQ standard Poor quality |
| Comments | 95% of answers to questionnaire from the contacted centers |

**Table I.** **Newcastle-Ottawa Quality Assessment table**

**Table J. Sensitivity analysis of ADPKD prevalence assuming different distribution of CKD stages in the population.**

|  | Measured | Hypothetical | | |
| --- | --- | --- | --- | --- |
|  |  | CKD 1-2  40% | CKD 1-2  50% | CKD 1-2  60% |
| Stage 1-2 of CKD (%) | 0.342 | 0.4 | 0.5 | 0.6 |
| Stage 3-5 of CKD (%) | 0.658 | 0.6 | 0.5 | 0.4 |
| Affected Subjects | 254 | 279 | 334 | 418 |
| prevalence  (in 10,000) | 3.62 | 3.97 | 4.76 | 5.96 |

**Table K. Type of variants (truncating, non truncating and in frame) of our cohort**

|  | PKD1 variants | PKD2 variants |
| --- | --- | --- |
| **Truncating (T)** |  |  |
| **Nonsense** | 5 | 2 |
| **Frameshift** | 5 |  |
| **Splicing** |  | 2 |
| **Atypical splicing** | 1 |  |
| **Intronic Deletion** | 1 |  |
| **Non truncating (NT)** |  |  |
| **Missense** | 6 |  |
| **Atypical splicing** | 1 |  |
| **Synonymous** | 1 |  |
| **In Frame** | 2 |  |

**Table L. Tables of logistic regression analysis.**

**Table M. Curve estimation cubic model summary and parameters results.**

**Fig A. The distribution of the cumulative risk to be affected according to age and differentiated by sex .**

**Fig. B Distribution of the Family Risk Score (affected/at risk subjects) in 99 families collected in the study.**

 The Family Risk Score has not been calculated in families of index cases without family history (sporadic cases).
